# *Bartonella rochalimae* in a flea collected from a *Mephitis macroura* in Sonora Mexico

**DOI:** 10.1007/s11686-024-00912-0

**Published:** 2024-08-30

**Authors:** Adriana M. Fernández-González, Angel Herrera-Mares, Fabiola Ramírez-Corona, Roxana Acosta, Gerardo Suzán

**Affiliations:** 1 Departamento de Etología, Fauna Silvestre y Animales de Laboratorio, Facultad de Medicina Veterinaria y Zootecnia, Universidad Nacional Autónoma de México, Ciudad de México, 04510 México; 2 Taller de Sistemática y Biogeografía, Departamento de Biología Evolutiva, Facultad de Ciencias, Universidad Nacional Autónoma de México, Ciudad de México, 04510 México; 3 Museo de Zoología Alfonso L. Herrera, Facultad de Ciencias, Universidad Nacional Autónoma de México, Ciudad de México, 04510 México

**Keywords:** *Bartonella*, Mexico, Flea, Carnivore

## Abstract

**Purpose:**

At least thirty species of wild carnivores have been recorded harboring *Bartonella*, and one of the most common pathogenic species infecting them is *Bartonella rochalimae*, which can cause endocarditis in humans and dogs. This bacterium can infect various mammals including wild carnivores, as well as ectoparasitic vectors such as fleas and ticks. Here we report the presence of *B. rochalimae*, in a *Pulex simulans* flea collected from a *Mephitis macroura* skunk in the municipality of Santa Cruz in Sonora, Mexico.

**Methods:**

Fleas were collected from a *M. macroura* in Sonora, Mexico, in October 2019. They were identified to species level and subsequently tested for the presence of *Bartonella* using molecular tools including conventional PCR, sequencing, and phylogenetic analysis.

**Results:**

A total of 10 *P. simulans* fleas (one male, nine females) were collected from the *M. macroura* skunk. The PCR and phylogenetic analysis indicated a prevalence of 10% (1/10) and a sequence clustered with the clade of *B. rochalimae*.

**Conclusions:**

We confirmed the presence of *B. rochalimae* in a *P. simulans* flea collected from a *M. macroura* skunk in the area of Santa Cruz, Sonora, Mexico. Based on our results and previous studies in northern Mexico, which are consistent, it is necessary to continue monitoring *Bartonella* in *M. macroura* skunks and their fleas, since they could be important reservoirs of this bacterium in northern Mexico.

The study of *Bartonella* in domestic carnivores has been prioritized by the well-known cat scratch disease caused by *Bartonella henselae*, however wild carnivores are also parasitized by this and other *Bartonella* species that compromise human health [1, 2]. At least thirty species of wild carnivores have been recorded harboring *Bartonella*, and one of the most common pathogenic species infecting them is *B. rochalimae* [2].

*Bartonella rochalimae* was described in 2007 from an American woman who exhibited symptoms of bacteremia, fever, and splenomegaly, after travel to Peru, where she had sustained multiple insect bites [3]. Subsequently human cases have been documented. In 2013 an outpatient with an unknown clinical course from Ethiopia presented *B. rochalimae*, and in 2024 a study reported a native patient from Guatemala who had contact with domestic animals and developed endocarditis [4, 5].

It is noteworthy that endocarditis has been documented not only in humans but also in animals. A retrospective study examining the clinical manifestations of infected dogs revelated that *B. rochalimae* is associated with infective endocarditis [6]. In addition, it appears that dogs may act as reservoirs of *B. rochalimae*, as evidence by a study in which dogs, cats, and guinea pigs were inoculated with a human isolate. This study observed that dogs exhibited high bacteremia for a period of 5–7 weeks, while cats did not show high levels of bacteremia and guinea pigs failed to detect infection [7]. Furthermore, there are documented cases of canine infection with *B. rochalimae* in various countries, including the United States, Iran, Mongolia, and Peru [8–11].

Additionally, *B. rochalimae* has also been detected in wildlife. In China, rodents of the species *Apodemus chevrieri* captured from 2020 to 2022 were found to be infected with *B. rochalima*e and the first record of an infection in wild carnivores was in *Vulpes vulpes* [12, 13]. Other carnivores such as *Canis aureus*, *Nyctereutes procyonoides viverrinus*, and *V. vulpes*, have also been found infected with this bacterium in Israel, Japan, and Austria, respectively [14–16]. In the Americas, *B. rochalimae* has been detected in carnivores, such as *Canis latrans*,* Mephitis mephitis*, *Procyon lotor*,* Urocyon cinereoargenteus*, and *V. vulpes* in the United States, as well as in several carnivore species in the north of Mexico, including *C. latrans*, *Mephitis macroura*, *M. mephitis*, and *Vulpes macrotis* [17, 18].

Additionally, *B. rochalimae* has also been detected in wildlife. In China, rodents of the species *Apodemus chevrieri* captured from 2020 to 2022 were found to be infected with *B. rochalima*e and the first record of an infection in wild carnivores was in *Vulpes vulpes* [12, 13]. Other carnivores such as *Canis aureus*, *Nyctereutes procyonoides viverrinus*, and *V. vulpes*, have also been found infected with this bacterium in Israel, Japan, and Austria, respectively [14–16]. In the Americas, *B. rochalimae* has been detected in carnivores, such as *Canis latrans*,* Mephitis mephitis*, *Procyon lotor*,* Urocyon cinereoargenteus*, and *V. vulpes* in the United States, as well as in several carnivore species in the north of Mexico, including *C. latrans*, *Mephitis macroura*, *M. mephitis*, and *Vulpes macrotis* [17, 18].

*Bartonella rochalimae* has been detected mainly in carnivore fleas and in some rodent fleas (*Ctenocephalides canis*, *Ctenocephalides felis*,* Echidnophaga gallinacea*, *Frontopsylla elatoides elatoides*, *Pulex irritans*, *Pulex simulans*,* Xenopsylla conforms conforms*,* Xenopsylla gerbilli minax* and *Xenopsylla* spp.), however, it has also been detected on fleas *Euhoplopsyllus glacialis* of *Sylvilagus audubonii* rabbits in United States, and on *Rhipicephalus sanguineus* and *Hyalomma dromedarii* ticks of Palestine dogs [18–28].

In North America, most investigations on *Bartonella* in wild carnivores and their associated fleas have been conducted in the United States [2]. However, few investigations on fleas and mammals in northern Mexico have recorded several pathogenic species including *B. rochalimae* [18, 29, 30]. López-Pérez et al. (2017) documented the presence *B. rochalimae* in fleas (*E. gallinacea*, *P. irritans*,* P*. *simulans*) collected from wild carnivores. Similarly, Zapata-Valdés et al. (2018) reported this bacterium in fleas *P. simulans* collected from *M. macroura*, in Sonora, Mexico. Due to the medical importance, the history of pathogenic species found, and the limited information in Mexico, it is prudent to continue monitoring *Bartonella* species that may be found infecting the fleas of wild carnivores. Here we report the record of *B. rochalimae* in a *P. simulans* flea collected from a *M. macroura* skunk in Sonora, Mexico.

In October 2019 in Santa Cruz, Sonora, Mexico, Tomahawk traps (Tomahawk Live Trap Inc, 30’’x 30’’ x 70’’) were placed to capture medium-sized wild mammals. A skunk *M. macroura* was caught and checked for fleas (Fig. 1). Fleas found were collected with the aid of a brush and tweezers and placed in microtubes with 90% ethanol at room temperature.

**Fig. 1:**
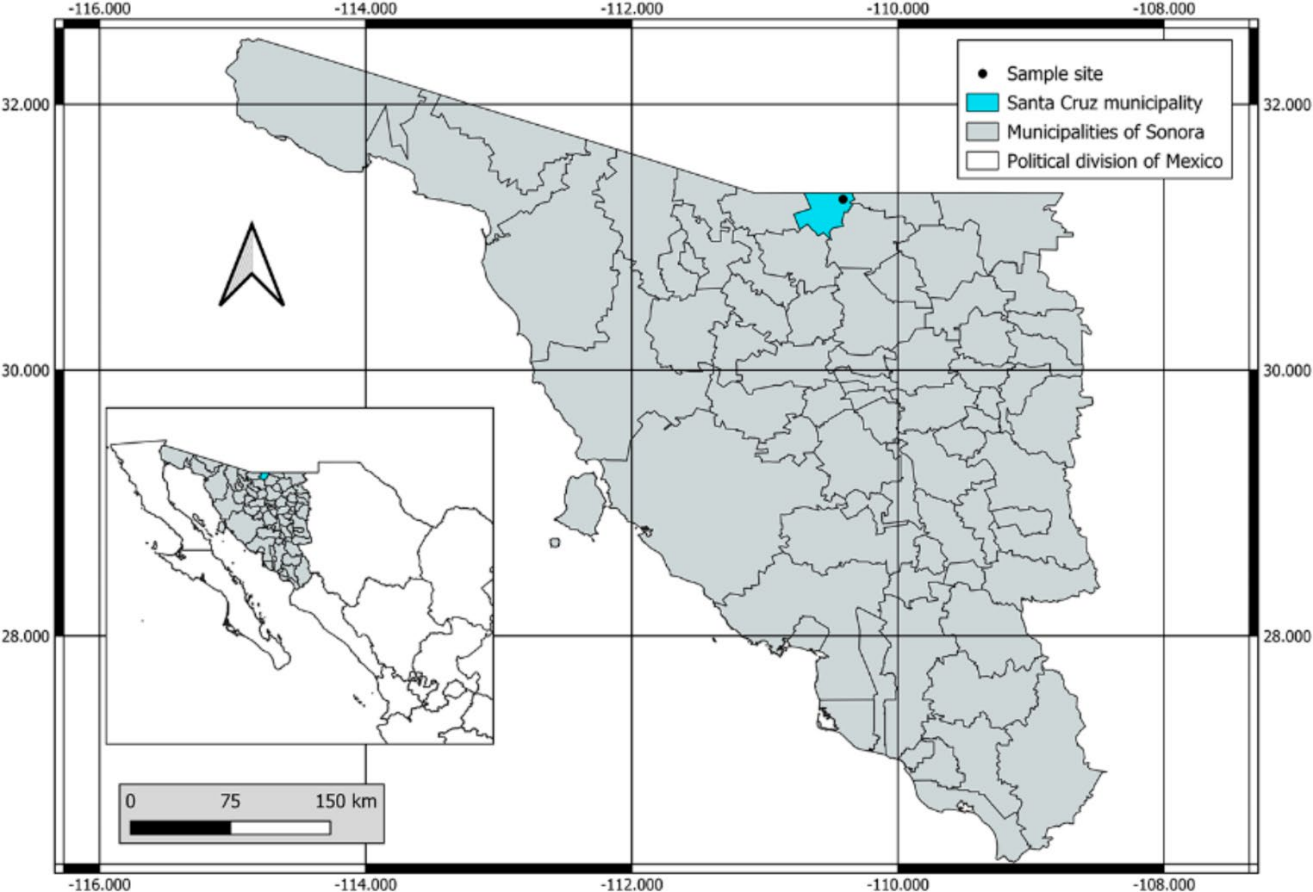
Sample site and municipalities of Sonora, Mexico (QGIS 3.32 program)

The flea identification was conducted subsequent to DNA extraction. Prior to extraction, each flea was punctured in the abdomen with a sterile entomological needle. Each individual flea was mounted on Canada balsam following Smit 1957 protocol in the Museo de Zoología Alfonso L. Herrera of the Facultad de Ciencias, Universidad Nacional Autónoma de México [31]. After mounting, each flea was identified to the species level based on the morphological characters, with particular emphasis on the genitalia, using an optical microscope (Olympus vanox-T microscope) and published taxonomic keys [32, 33]. As the morphological characters of the genitalia of *P. simulans* and *P. irritans* females are very similar, the presence of males in the same collection may be prove beneficial, as male genitalia are clearly different. In this instance, given that nine female fleas and one male flea were found, it was assumed that they all belonged to the *P. simulans* species.

DNA extraction from each flea was performed using the DNeasy Blood and Tissue kit (Qiagen^®^) following the supplier’s protocol. For *Bartonella* detection, PCR was performed by amplifying 767 bp of the *gltA* gene using previously described primers (CS443f: 5′ GCTATGTCTGCATTCTCTCTATCA 3′ and CS1210r: 5′ GATCYTCAATCATTTCTTTCCA 3′) [34]. Conventional PCR was performed in a final volume of 25 µl containing 12.5 µl Top Taq^®^ Master Mix, 5 µl nuclease-free water, 2.5 µl CoralLoad buffer, 2.5 µl DNA and 1.25 µl (10 µM) of each primer under the following conditions: initial denaturation (2 min 94 °C), followed by 45 cycles at 94 °C for 30 s, 48 °C for 1 min, and 72 °C for 1 min, and a final extension of 72 °C for 7 min. After PCR, aliquots (5 µl) of individual amplicons were subjected to 1% agarose gel electrophoresis at 100 V for 50 min, subsequently fragments were visualized with ethidium bromide under ultraviolet light. Upon visualization, an amplicon corresponding to *Bartonella* sp. was purified and sequenced at the Instituto de Biología (Universidad Nacional Autónoma de México). To corroborate the identity of the amplification, the sequence obtained was visualized with the Chromas 2.6.6 program and analyzed with the BLAST algorithm to compare it with those deposited in GenBank [35]. Subsequently, MAFFT version 7 was used to align our sequence and those obtained from GenBank. A phylogenetic tree was reconstructed by Maximum Likelihood using the program RaxMLGUI 2.0.10 under the TIM2 + G nucleotide substitution model, selected from the Bayesian information criterion in the RaxMLGUI, with a Bootstrap value based on 1000 pseudoreplicates [36].

The results of this study showed that the flea *P. simulans* was the only species collected from the *M. macroura*. According to the PCR, we observed a prevalence of 10% (1/10) and the BLAST of the sequence obtained from this *P. simulans* female flea, showed a percentage of identity of 99.61% (509/511; Query cover 100%) with *Bartonella rochalimae* (GenBank accession number OQ436436), referred as *Bartonella* sp. in [37]. Interestingly, our sequence (PP717818) was clustered to the clade formed by *B. rochalimae*, and other sequences obtained from a human (FN645459), domestic and wild carnivores (DQ676484, CP019785, CP019782, CP019786), and *Pulex* sp. fleas collected from wild carnivores (OQ436436, OQ436435) (Fig. 2).

**Fig. 2:**
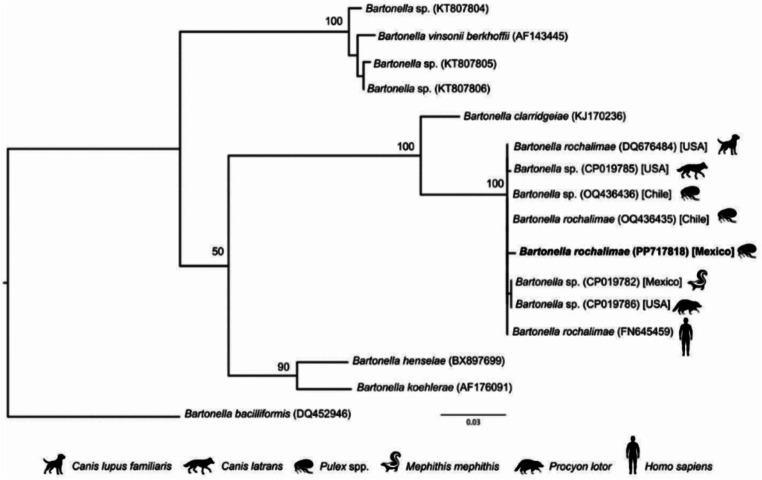
Phylogenetic tree showing *Bartonella* sequences obtained from a *P. simulans* flea collected from a wild *Mephitis macroura* in Santa Cruz México, and selected sequences from the GenBank. Each sequence has its GenBank accession number, and our sequence is indicated with boldface

The results of our study align with those of previous research conducted in northern Mexico. A study conducted in the same area detected *B. rochalimae* in *P. simulans* fleas collected from a *M. macroura*, with a reported prevalence of 14% (2/14), however, the work mentioned above does not have sequences deposited in GenBank [30]. Consequently, this study provides the first deposited sequence of *B. rochalimae* in Sonora state. In contrast, a study conducted in the state of Chihuahua, situated adjacent to Sonora, has identified *P. simulans* fleas as the most abundant in carnivorous species, including *C. latrans*,* M. macroura*,* M. mephitis*, and *U. cinereoargenteus*. Additionally, these fleas exhibited the highest infection rates for *B. rochalimae* and *Bartonella vinsonii berkhoffii* [18]. It can thus be postulated that *P. simulans* fleas may play an important role in the transmission of *Bartonella* species in wild carnivores such as *M. macroura* in northern Mexico. *Mephitis macroura* forages for food by consuming insects, fruits, small vertebrates, and bird eggs. However, they also scavenge in garbage dumps, where they and their fleas may come into contact with domestic animals and/or humans, potentially transmitting *B. rochalimae* or other pathogens that cause zoonotic diseases (e.g., Chagas or rabies) [38, 39].

The present study was constrained by the limited number of fleas collected from a single *M. macroura* and the exclusive utilization of a single gene (*gltA*). It is recommended that multiple genes be employed for the differentiation of *Bartonella* species. However, the *gltA* gene is one of the most reliable and widely utilized [40, 41]. Notwithstanding the aforementioned limitations, our study represents the first report of a *B. rochalimae* sequence from a *P. simulans* flea collected from a *M. macroura* in Santa Cruz, Sonora, Mexico. We suggest continuing *Bartonella* monitoring in *M. macroura* and its fleas, as they may serve as important and key reservoirs for the transmission of *B. rochalimae* at the interface between wildlife, domestic carnivores, and humans in northern Mexico.
